# Metformin induces protein acetylation in cancer cells

**DOI:** 10.18632/oncotarget.17829

**Published:** 2017-05-11

**Authors:** Ales Vancura, Ivana Vancurova

**Affiliations:** Department of Biological Sciences, St. John's University, NY, USA

**Keywords:** AMPK, metformin, acetyl-CoA, protein acetylation, ovarian cancer

**Figure 1:**
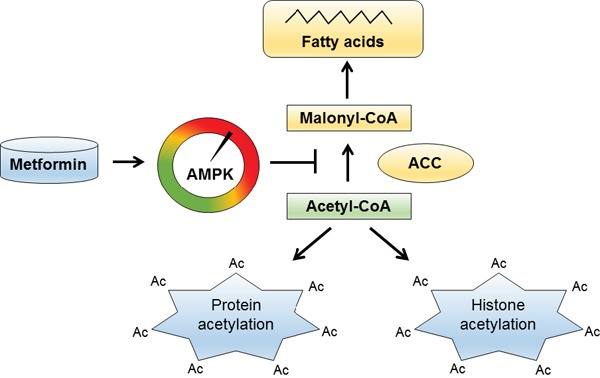
Metformin activates AMPK and induces histone acetylation In prostate and ovarian cancer cells, metformin activates AMPK, which phosphorylates and inactivates ACC. This increases nucleocytosolic level of acetyl-CoA due to the decreased conversion of acetyl-CoA into malonyl-CoA. The nucleocytosolic acetyl-CoA is then used for acetylation of histones and other proteins.

AMP-activated protein kinase (AMPK) is an energy sensor and master regulator of metabolism. AMPK functions as a fuel gauge monitoring systemic and cellular energy status. Activation of AMPK occurs when the intracellular AMP/ATP ratio increases and leads to a metabolic switch from anabolism to catabolism. Metformin, widely used for diabetes type 2 treatment, activates AMPK by inhibiting mitochondrial respiratory chain complex I, leading to decreased ATP production and increased AMP/ATP ratio. We have recently shown that AMPK activation with metformin affects acetyl-CoA homeostasis and induces protein acetylation in cancer cells [1] (Figure 1).

The recent interest in the use of metformin and other AMPK agonists to support cancer prevention and treatment is based on clinical studies that have shown that the use of metformin is associated with decreased cancer incidence in diabetic patients [2]. The mechanism of metformin function in diabetes treatment consists of decreasing glucose production by gluconeogenesis in the liver, through inhibition of the mitochondrial respiratory chain complex I. The decrease in mitochondrial ATP production results in AMPK activation; however, the AMPK activation does not seem to be required for the anti-diabetic effect of metformin [3]. Untreated diabetes type 2 is associated with an increased cancer risk, attributed mostly to the growth-promoting effect of chronically elevated plasma glucose and insulin levels. The mechanism of the metformin’s anti-tumor effect is not completely understood. It appears that metformin inhibits tumor growth through both AMPK-independent and AMPK-dependent mechanisms. The AMPK-independent mechanism has been attributed to the decreased glucose and insulin blood levels. The AMPK-dependent mechanism of metformin is mediated through the inhibition of mTORC1 signaling and NFκB pathway [2, 3]. 

In addition to the above effects, AMPK activation increases histone acetylation [1]. Active transcription generally correlates with increased acetylation of promoter histones. Histone deacetylase (HDAC) inhibitors have been developed for cancer treatment with the aim of increasing histone acetylation and transcription of tumor suppressor genes, which are silenced in cancer cells [4]. In general, HDAC inhibitors increase histone acetylation, expression of p21 and pro-apoptotic genes, and induce apoptosis. Our recent results have shown that similarly to HDAC inhibitors, activation of AMPK increases histone acetylation within the p21 promoter, and p21 expression. 

How does metformin increase histone acetylation? Histone acetylation depends on intermediary metabolism for supplying acetyl-CoA in the nucleocytosolic compartment [5]. The nucleocytosolic acetyl-CoA is a critical precursor of several anabolic processes including *de novo* synthesis of fatty acids. Acetyl-CoA carboxylase (ACC) catalyzes the carboxylation of acetyl-CoA to malonyl-CoA, the first and rate-limiting reaction in the *de novo* synthesis of fatty acids. The ACC activity affects the concentration of nucleocytosolic acetyl-CoA. Attenuated expression of yeast ACC increases global acetylation of chromatin histones, and alters transcriptional regulation [6]. Direct pharmacological inhibition of ACC in human cancer cells also induces histone acetylation [1]. ACC is phosphorylated and inhibited by AMPK. In yeast, inactivation of SNF1, the budding yeast ortholog of mammalian AMPK, results in increased ACC activity, reduced pool of cellular acetyl-CoA, and globally decreased histone acetylation [7]. Activation of AMPK with metformin or with the AMP mimetic AICAR increases the inhibitory phosphorylation of ACC, and decreases the conversion of acetyl-CoA to malonyl-CoA, leading to increased protein acetylation and altered gene expression in prostate and ovarian cancer cells (Figure 1). Since AMPK activation requires LKB1 kinase, metformin does not induce protein acetylation in LKB1-deficient cells [1]. 

These results indicate that AMPK regulates the availability of nucleocytosolic acetyl-CoA for protein acetylation and that AMPK activators, such as metformin, have the capacity to increase protein acetylation and alter patterns of gene expression, further expanding the plethora of metformin’s physiological effects. Metformin displays anti-proliferative and pro-apoptotic properties towards cancer cells; however, the underlying mechanisms are not yet fully understood. The effect of metformin on protein acetylation and transcriptional regulation may represent one of these mechanisms, and may provide a rationale for the development of novel combination anti-cancer therapies involving metformin and other AMPK agonists.
